# Autophagy modulation: a prudent approach in cancer treatment?

**DOI:** 10.1007/s00280-018-3669-6

**Published:** 2018-09-04

**Authors:** Eleanor Bishop, Tracey D. Bradshaw

**Affiliations:** School of Pharmacy, Centre for Biomolecular Sciences, University of Nottingham, University Park Nottingham, NG7 2RD UK

**Keywords:** Autophagy, Tumourigenesis, Tumour suppression, Tumour promotion, Induction, Inhibition

## Abstract

Autophagy is a tightly controlled process comprising lysosomal degradation and recycling of cellular proteins and organelles. In cancer, its paradoxical dual role of cytoprotection and cytotoxicity is context-dependent and controversial. Autophagy primarily acts as a mechanism of tumour suppression, by maintenance of genomic integrity and prevention of proliferation and inflammation. This, combined with immune-surveillance capabilities and autophagy’s implicated role in cell death, acts to prevent tumour initiation. However, established tumours exploit autophagy to survive cellular stresses in the hostile tumour microenvironment. This can lead to therapy resistance, one of the biggest challenges facing current anti-cancer approaches. Autophagy modulation is an exciting area of clinical development, attempting to harness this fundamental process as an anti-cancer strategy. Autophagy induction could potentially prevent tumour formation and enhance anti-cancer immune responses. In addition, drug-induced autophagy could be used to kill cancer cells, particularly those in which the apoptotic machinery is defective. Conversely, autophagy inhibition may help to sensitise resistant cancer cells to conventional chemotherapies and specifically target autophagy-addicted tumours. Currently, hydroxychloroquine is in phase I and II clinical trials in combination with several standard chemotherapies, whereas direct, deliberate autophagy induction remains to be tested clinically. More comprehensive understanding of the roles of autophagy throughout different stages of carcinogenesis has potential to guide development of novel therapeutic strategies to eradicate cancer cells.

## Introduction

Autophagy is a highly conserved process of catabolism, which recycles and degrades intracellular components, primarily as a mechanism of cellular survival [1]. It was first revealed in 1949 by Christian de Duve following the discovery of lysosomes and their functions [2]. In eukaryotic cells, there are three types of autophagy known as macroautophagy, microautophagy and chaperone-mediated autophagy [3]; the majority of our understanding is of macroautophagy (referred to as autophagy from here onwards). Basal autophagy is fundamental to cellular (metabolic and genetic) homeostasis, involved in quality control of proteins and organelles in the cellular environment [4]. In stressful environments and under starvation conditions, autophagy can also be induced to sustain metabolic demands, by producing substrates required for cell survival, and maintain genomic integrity.

**Fig. 1:**
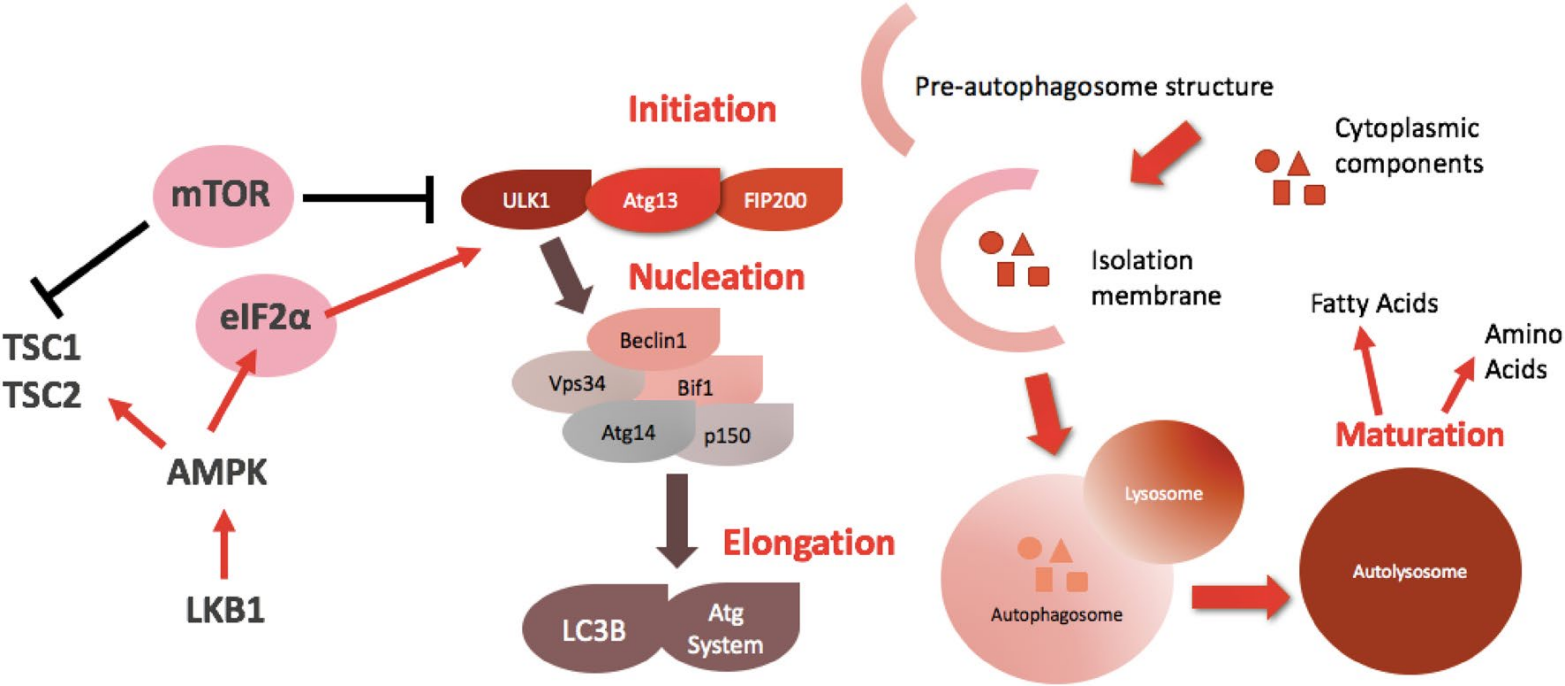
The genetic background and steps of autophagy. ULK1 phosphorylation status is critical to autophagy initiation. Once activated, it forms a complex with Atg13 and FIP200, which further activates beclin1. Beclin1 activation completes initiation and is central to the nucleation process. Subsequently, LC3B complexes with the Atg system, which elongates the isolation membrane to form the autophagosome. Lysosomal fusion is central to the maturation process to produce the autolysosome. This is responsible for the degradation and recycling of intracellular components to substrates such as amino acids and fatty acids, which can be used for cell growth. mTOR and eIF2a are the two main cellular sensors which control the status of autophagy: mTOR inhibits the process, whereas eIF2a activates it

Autophagy is a tightly controlled process, influenced by multiple signalling pathways. Autophagy-related genes (*Atg* genes) orchestrate the formation of the autolysosome, through a series of sequential steps: initiation, nucleation, elongation and maturation [5, 6]. Figure 1 highlights some of the key genes and signalling molecules involved in the process and depicts the process of phagosome formation and lysosomal degradation. The mammalian target of rapamycin (mTOR), a downstream protein in the phosphatidylinositol-3-kinase (PI3K) cascade, is the main inhibitor of autophagy, preventing its execution when nutrients are abundant [7]. Conversely, the initiation factor eiF2a senses nutrient depletion and activates the process to provide cellular substrates including fatty acids and amino acids [8].

Autophagy has dual, apparently paradoxical roles in cytoprotection and cytotoxicity. Preventing the accumulation of damaged proteins or organelles protects the cell from oxidative stress and subsequent DNA damage, however, if autophagy is extensive and excessive, this can lead to cellular destruction termed type II programmed cell death [6]. Autophagy activation and dysregulation has implications in disease pathogenesis including type II diabetes, cardiovascular and neurological disorders, microbial infections and cancer [6]. The role autophagy plays in cancer is controversial with evidence that autophagy plays a part in both suppressing and promoting tumourigenesis. More comprehensive understanding of its functions could provide a novel approach to cancer treatment, by providing new strategies to modulate this process in a therapeutic arena.

## Autophagy: a role in tumour suppression

A large body of evidence suggests autophagy primarily acts as a tumour suppressive mechanism. For example, Ras-driven epithelial tumourigenesis is suppressed by autophagy by limiting accumulation of reactive oxygen species (ROS) [9]. Commonly mutated oncogenes repress autophagy, whereas tumour suppressors work to activate the process [10]. Beclin1 is a mammalian autophagy-related protein, key in the initiation and nucleation processes [10, 11]; *beclin-1* gene is monoallelically deleted in 40–75% of breast, ovarian and prostate cancers and has reduced expression in human breast carcinoma lines [11, 12]. Additionally, heterozygous mice are prone to the development of spontaneous tumours including lung, liver carcinomas and lymphomas [11]. Reduced expression of beclin1 protein has also been shown in a variety of brain tumours, cervical and hepatocellular carcinoma and colorectal cancer cell lines [13–16]. Unsurprisingly, beclin1 has been proposed as a candidate tumour suppressor gene, with its disruption likely to be ‘mechanistically key’ in tumourigenesis [1].

Deletion of *Atg 5* and *Atg7* causes spontaneous formation of benign liver tumours [17], further suggesting a role for autophagy in tumour suppression. Atg5 and beclin 1 have been shown to act as ‘guardians’ of the cellular genome, by preventing DNA damage, aneuploidy and amplification [1, 18]. Furthermore, tumours with mono- or biallelic loss of these genes display increased tumourigenicity [18]. Autophagy-deficient yeast display a similar phenotype with an increase in mitochondrial mutations; this suggests that autophagy turnover acts to eradicate cells possessing DNA damage, preventing persistence of mutations [19, 20]. Although not fully understood, it provides evidence that autophagy dysregulation may cause (or at least contribute to) genomic instability, acquisition of which drives evolution of multiple cancer hallmarks enabling tumour promotion [21].

**Fig. 2:**
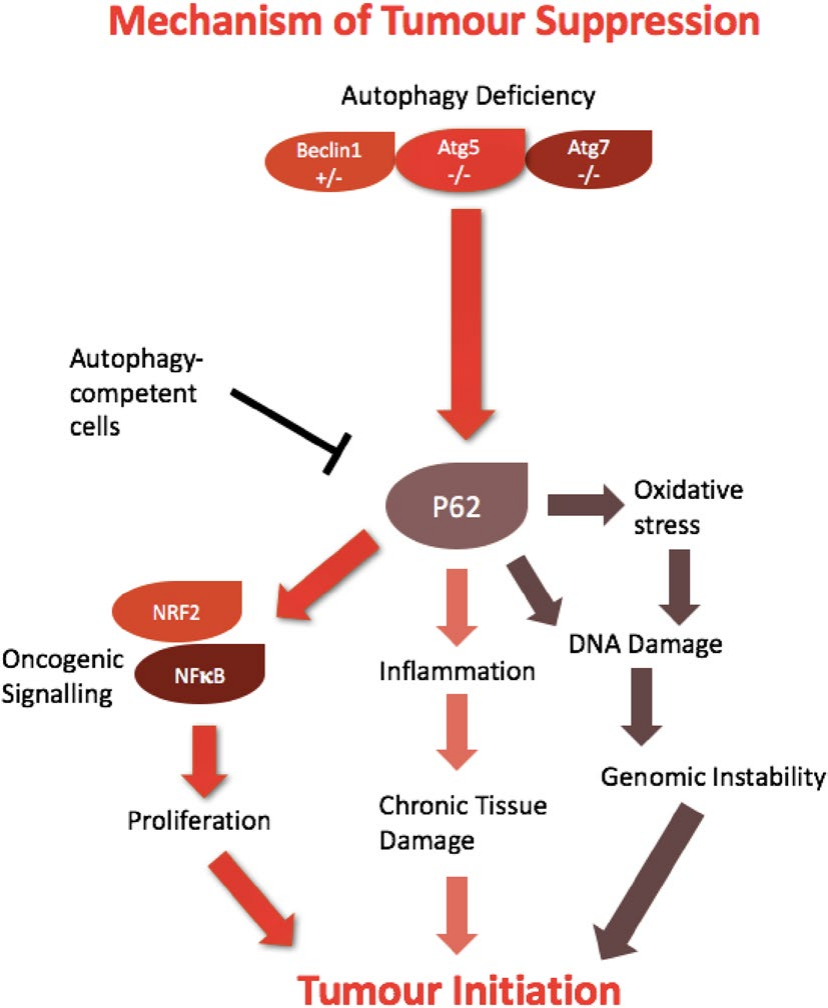
Autophagic mechanisms of tumour suppression. Autophagy deficiency can cause the accumulation of P62 which subsequently drives tumour initiation. P62 can activate oncogenic signalling pathways to stimulate cellular proliferation, as well as increasing oxidative stress and inflammation. In turn, this can lead to genomic instability and chronic tissue damage, promoting spontaneous tumour formation

The mammalian proteins p62 and NBR1 selectively undergo autophagic degradation and can act as cargo receptors, or adaptors for autophagy of ubiquitinated substrate targets [22]. A key role for p62-mediated autophagy has been implicated in tumourigenesis; in a state of autophagic-deficiency, p62 may accumulate (Fig. 2) [4]. P62 activates oncogenic signalling cascades involving NFkB and NRF2, both of which are able to drive tumour formation [23, 24]. The role of p62 has also been explored in mouse models, whereby accumulation is coupled with a state of chronic inflammation and tissue damage, for example, as shown in the liver and pancreas [23, 25]. p62 also promotes tumour initiation by causing mitochondrial defects, increasing oxidative stress and subsequent DNA damage [4]. This presents a setting whereby autophagy can work to diminish metabolic stress and prevent pro-tumourigenic events from occurring.

## Autophagy: a role in immune surveillance

As alluded to, autophagy also has a tumour suppressive role in established cancer cells. Autophagy is critical for optimal immune function, indeed many studies have shown autophagy to be essential for the recruitment of immune cells to the tumour bed, including dendritic cells and cytotoxic T lymphocytes, which are key in the stimulation of anti-cancer immune responses [26, 27]. Indeed, inhibition of autophagy has been shown to lead to inhibition of the immune system, tumour immune escape and development [28]. Autophagy generates extracellular ATP which is thought to be the mechanism behind immune cell recruitment. Further supporting this thesis, autophagy inhibition promotes ATP degradation to adenosine, which facilitates accumulation of anti-immune T regulatory cells [29]. Immune competency has been shown to increase sensitivity in both radiotherapy and chemotherapy-treated cancer cells [27, 30, 31]. For malignant cells to survive and tumours to progress require escape from immune detection. Novel anti-cancer regimens are being explored, involving autophagy induction that re-establishes immune surveillance. Nutrient deprivation or caloric restriction mimetics reduce lysine acetylation of cellular proteins, triggering autophagy. Short-term fasting, or autophagy-inducing caloric restriction mimetics have been shown to enhance chemotherapeutic efficacy (of mitoxantrone and oxaliplatin) [29, 31].

Thus, it may be argued that activating autophagy might be a relevant clinical objective to enhance anti-tumour immune surveillance, and potentially increase the efficacy of current conventional therapies.

## Cytotoxic autophagy

Autophagy also acts in a tumour suppressive manner via its capacity to induce cell death. Autophagic cell death is referred to as type II programmed cell death and is characterised by the presence of cytoplasmic vacuolisation, with increased autophagic flux. Unlike apoptosis, autophagic cell death occurs independently of caspase activity [6, 26]; it lacks the tissue inflammatory response associated with necrosis, which may otherwise promote tumour formation [32]. Autophagic cell death is commonly seen throughout embryogenesis [33, 34], but is not restricted to developmental stages of life. In differentiated tissues, tumour suppressive autophagy has been observed: dendrogenin A (DDA), a newly discovered cholesterol metabolite found in mammals, possesses tumour cell differentiation and tumour suppressive properties in breast cancers, melanoma and acute myeloid leukaemia (AML), including primary AML patient samples [34, 35]. Metabolic studies demonstrated reduced DDA levels in cancer cells and human tumours compared to normal cells and tissues [35, 36]. In vitro and in vivo, DDA triggers tumour cytotoxic autophagy by binding to the liver x receptor (LXR), a nuclear receptor, and by inducing the transcriptional expression of pro-autophagic factors such as LC3b, Nur77, NOR1 and TFEB [34]. Interestingly, TFEB is a transcription factor and master gene-controlling lysosomal biosynthesis and autophagy. Moreover, by inhibiting the D8D7I subunit of the cholesterol epoxide hydrolase enzyme (ChEH), DDA induces accumulation of sterols which contribute to increased formation of lysosomes—essential components of the autophagy machinery [34]. Thus, the nuclear receptor LXR has been identified as an essential molecular target activating lethal autophagy in cancers, and the combined action of DDA on LXR and D8D7I most likely contributes to its high efficacy by triggering sustained lethal autophagy, which is not observed with prototypical ChEH/D8D7I inhibitors or other LXR agonists [36, 37]. The anti-tumour activity and low-toxicity of DDA support the clinical development of DDA.

**Fig. 3:**
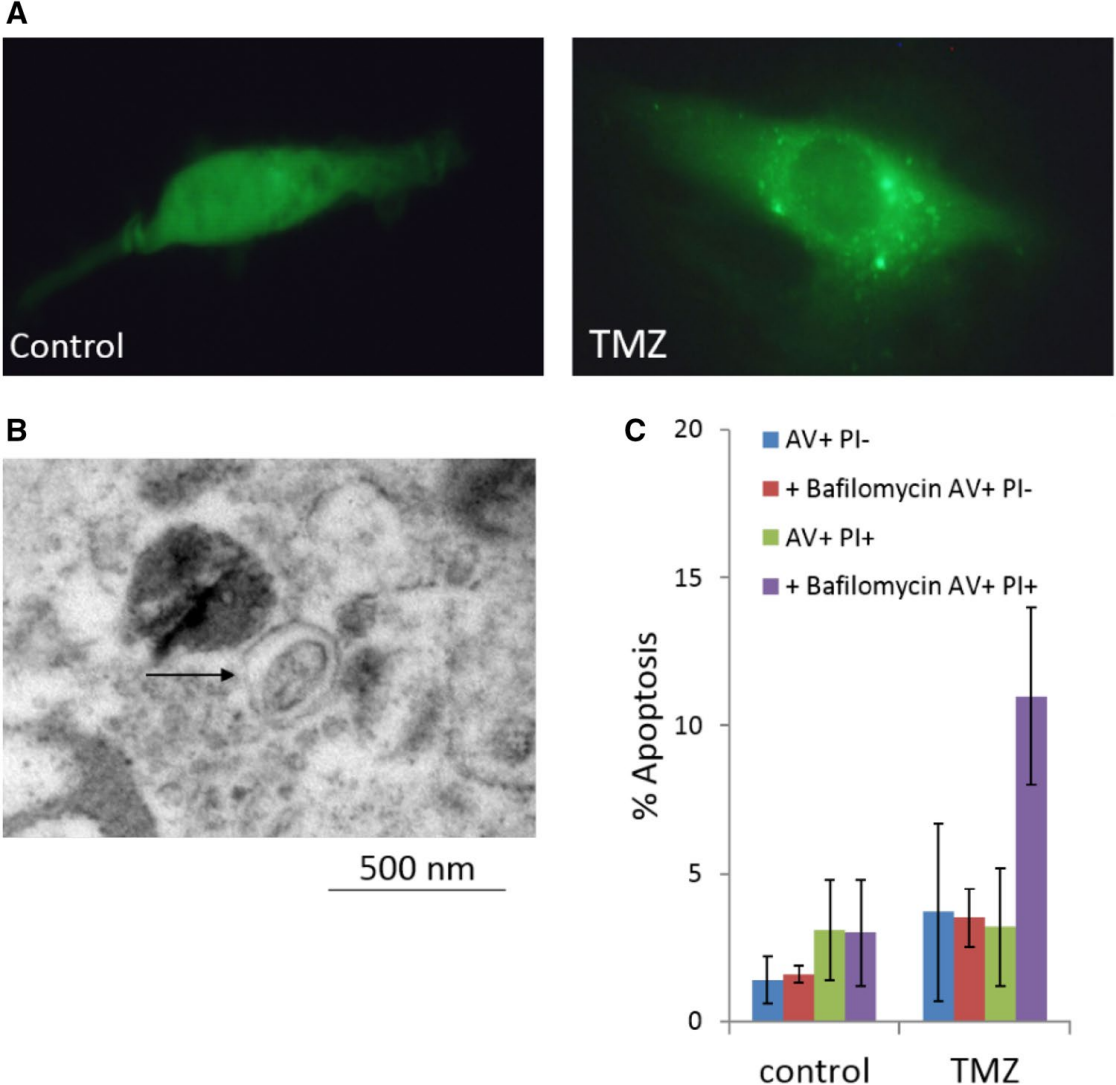
Induction of autophagy by temozolomide in U87MG GBM cells (100 µM; 72 h). **a** Accumulation of acidic vesicular organelles; **b** detection of autophagosomes—double-membrane vacuoles; **c** inhibition of temozolomide-induced autophagy resulting in cell death by apoptosis [42]

A variety of clinical anti-cancer therapies have been reported to induce cytotoxic autophagy including resveratrol [38], arsenic trioxide [39], and tamoxifen [6, 40], suggesting autophagy directly contributes to the cytotoxic effects of these drugs. However, the relationship between steroid biogenesis, tamoxifen and autophagy are complex—as will be discussed, autophagy may contribute to tamoxifen-resistance. In tamoxifen-resistant MCF-7 (TAMRMCF-7) cells, caspase-independent autophagic cell death has been induced by the histone deacetylase (HDAC) inhibitor suberoylanilide hydroxamic acid (SAHA). Significantly decreased HDACs 1, 2, 3, 4, 7 and enhanced acetylation of histones 3 and 4 accompanied expression of autophagic markers LC3-II and beclin-1 [41]. In mice-bearing TAMRMCF-7 xenografts, SAHA significantly reduced tumour growth without side effects. Authors concluded that SAHA-mediated autophagic cell death offers a promising strategy for treatment of tamoxifen-resistance breast cancer. Finally, the alkylating agent temozolomide, used in standard of care chemotherapy for glioblastoma multiforme (GBM) has been shown to induce autophagic cell death in apoptosis-resistant GBM models (Fig. 3) [42]. Subsequent inhibition of the autophagy-related genes beclin1 and Atg5 has been shown to reduce cell death, further supporting this cytotoxic role in a cancerous setting [43].

## Autophagy induction

Although there have currently been no attempts to directly induce autophagy in cancer models, modulation of this process could comprise a new anti-cancer strategy in the future [44]. The role of autophagy in preventing tumour initiation has been well-researched and forms an opportunity to harness this process. Autophagy induction could be beneficial in deficient or mutated pre-cancerous cells, by way of preventing tumour initiation. Autophagy induction could help to reduce the accumulation of p62, particularly in tissues where chronic inflammation is known to be an evolutionary pre-cursor to cancer. In the future, p62 could be used as a biomarker to indicate autophagy deficiencies and identify patients who may benefit from autophagy induction [45]. Additionally, autophagy confers immunogenic properties in existing tumour cells, thus it is pertinent to test whether induction can stimulate anti-cancer immune responses and increase cancer cell sensitivity to conventional therapies.

To summarise, it is of clinical interest to investigate further whether autophagy induction can be exploited to prevent tumour initiation, reduce tumourigenicity in established cancers, enhance efficacy, or mediate cytotoxicity of chemotherapy. Observations have also indicated that induction of autophagic cell death may provide an attractive therapeutic strategy, particularly in cancer cells able to evade apoptosis, which is a common hallmark of many cancers [11].

A deeper understanding of the process of lethal autophagy, and how molecular mechanisms interact with other cell death pathways, will be crucial in the development of effective strategies to accomplish cancer cell toxicity.

## Controversy

Although autophagy has been widely implicated as a form of cell death, controversy remains concerning the extent of its actions. It is possible that autophagy is merely associated with dying cells and not a direct cause of the toxic fate. Indeed, autophagic tumour cell death has only been reported in very few circumstances and instead has been shown to activate alternative mechanisms executing cell death [33, 46]. It has been postulated that autophagic association could represent an adaptive response, a failed attempt at cell survival [6]. Targeting this process in vivo could prove highly challenging, as multiple checkpoints prevent type II cell death from occurring [44]. Autophagy has also been described as a mechanism of tumour promotion, thus fully understanding the context in which autophagy occurs will be critical before developing any induction-based therapies, which could conversely support tumourigenicity.

## Autophagy: a role in tumour promotion

Although autophagy has been shown to act in a tumour suppressive manner, its role is context-dependent; once tumours are established, they can exploit this process to enable survival. This paradoxical change from tumour suppressor to tumour promoter has been termed the ‘autophagy switch’ [47]. Cancer cells can use autophagy to survive the hostile metabolic microenvironment, utilising autophagic substrates to sustain tumour growth and maintain cellular fitness.

Autophagy promotes tumourigenesis by providing an escape mechanism to cellular stresses. Within the tumour microenvironment, cancer cells experience harsh conditions including hypoxia and acidity. Cytoprotective autophagy provides a mechanism to promote cancer cell survival, removing otherwise toxic proteins and providing cellular substrates to sustain growth [46]. Hypoxic regions have shown elevated levels of autophagy, recycling organelles such as ribosomes and mitochondria, allowing resulting catabolites to fuel biosynthesis and energy metabolism—maintaining cancer cell viability [6, 48, 49].

Autophagy is robustly activated in tumour cells by cancer-associated stressors such as growth factor deprivation and hypoxia. In the majority of cases, autophagy induction promotes survival in response to stress (as will be exemplified); survival by autophagy becomes dangerous if apoptosis is disabled—resulting in quiescence/dormancy of tumour cells. Hypoxia and glucose deprivation (conditions rife within the tumour microenvironment) upregulate autophagy—enabling survival. Autophagosomes are most evident in tumour cells of hypoxic regions, and deletion of autophagy-regulating genes has been shown to result in tumour cell death especially in hypoxic regions, strongly indicating that cancer cells exploit autophagy to survive and promote tumourigenesis [18]. A role for autophagy in promotion of a carcinoma fate is further supported by observations of malignant adenoma formation in autophagy-competent cells [17]. Other mouse models have demonstrated the role of autophagy in the development of a carcinoma phenotype and have shown autophagy inhibition to reduce tumour volume [6, 17]. A link between autophagy and p53 suppression has been proposed, which could underlie this tumour-promoting background in some circumstances [50]. Robust evidence supports the argument that inhibition of autophagy, as a therapeutic strategy could serve to reduce tumour aggression and promote a more benign, ‘treatable’ state [4].

## Autophagy addiction

**Fig. 4:**
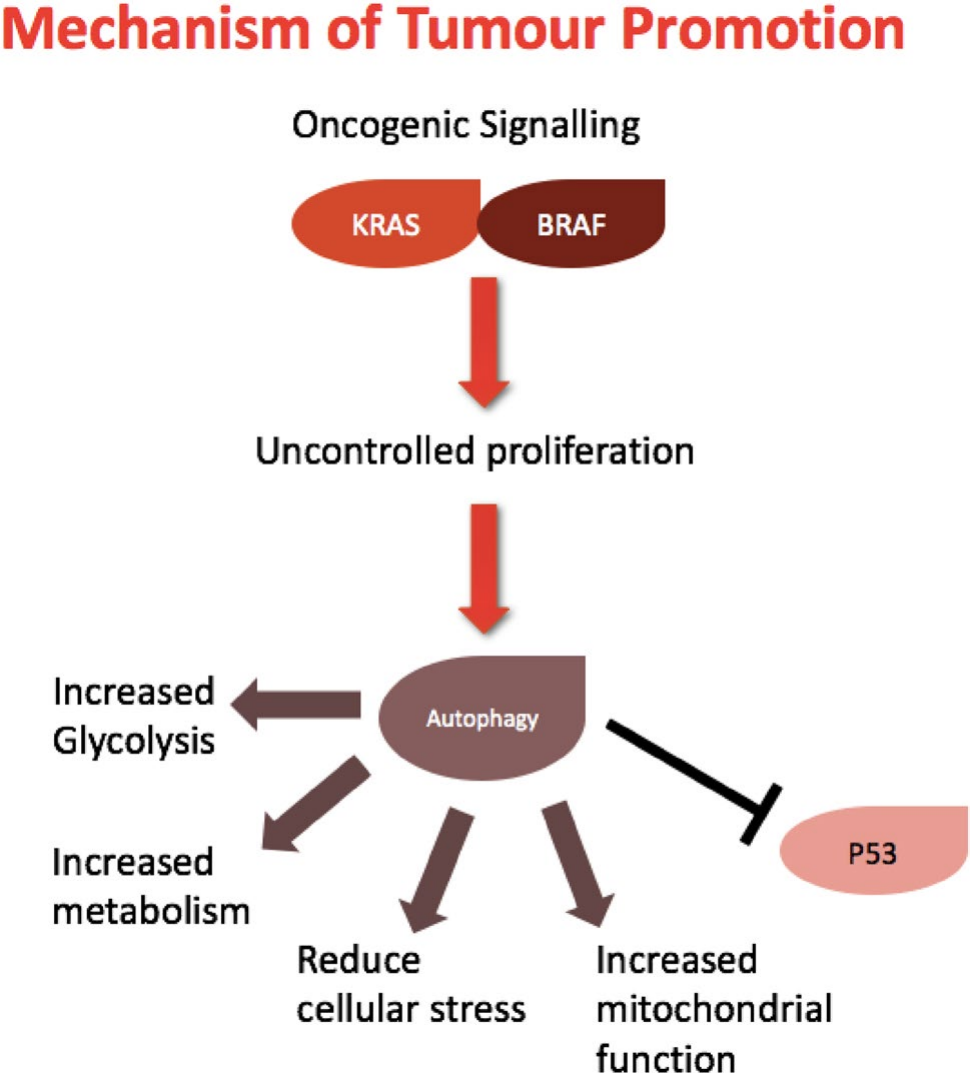
Autophagic mechanisms of tumour promotion. Increased oncogenic signalling present in established tumours can exploit autophagy to sustain tumour growth and survival. Uncontrolled proliferation leads to increased metabolic demands and cellular stress; autophagy can provide cells with substrates for growth, inhibit tumour suppressive functions of p53 and increase metabolic and glycolytic functions of cancer cells

Certain tumour types have shown elevated levels of autophagy, thus ‘autophagy addiction’ has been proposed as a form of tumour maintenance or promotion [4]. Tumours harbouring Ras and RAF mutations lie in this category. Ras-mutated tumours have shown an increased dependency on the autophagic process in vitro and in vivo, and knockdown of autophagy-related genes *Atg5* and *Atg7* limited tumour growth [6, 51, 52]. Ras mutations can cause a metabolic depletion of cellular energy sources; thus, autophagy can enable cell survival by preserving mitochondrial integrity and directly providing substrates required for cell growth [53]. It has been suggested that the role of autophagy in this setting is intrinsically connected to the status of p53. Autophagy has been shown to inhibit this tumour suppressor gene to accelerate tumourigenesis of mutant Ras tumours [51, 54]. Autophagy addiction has also been implicated in a specific metabolic context; autophagy-competent cells have an increased glycolytic capacity and show elevated levels of glycolysis. Inhibition of autophagy attenuates proliferation and reduces this metabolic alternative [55]. Together, these autophagic activities facilitate Ras-driven transformation, summarised in Fig. 4.

Pancreatic tumours typically harbour Ras mutations and frequently show constitutive levels of autophagy [6]. Genetic and chemical modes of autophagy inhibition have caused significant tumour regression of pancreatic cancer cells in vitro [56]. RAF-mutated tumours have also demonstrated increased dependency on autophagy to sustain tumour growth [57]. V600E mutations, characteristic of melanoma, exploit autophagy to preserve mitochondrial function and reduce oxidative stress [44, 57]. Autophagy competency in tumour cells is likely to confer a selective advantage, providing a mechanism of survival to withstand cellular stresses. Ras or RAF-driven tumours typically have a poor prognosis due to their aggressive nature [52]; thus, fully understanding the role of autophagy-dependency in this context could provide a novel therapeutic strategy to target these tumours.

## Autophagy-mediated drug resistance and cancer cell survival

In addition to its role in tumour promotion and survival in hostile environments, autophagy has also been implicated in resistance to conventional anti-cancer therapies, another form of stress imposed on cancer cells [54]. Returning to tamoxifen, autophagy`s role in resistance to this (and other) selective oestrogen receptor modulators (SERMs) has been revealed. Tamoxifen is commonly used in the treatment of oestrogen receptor positive (ER+; the most frequent subtype) breast cancer; however, treatment failure resulting from acquired resistance to tamoxifen is often observed. De Medina et al. [58] established that sterol-dependent macroautophagy induced by tamoxifen may be associated with cell survival rather than cytotoxicity; inhibition of autophagy (by 3-methyl adenine or bafilomycin) sensitised cells to tamoxifen and other SERMs [33]. Tamoxifen competitively binds not only to the ER, but also to microsomal antioestrogen binding sites comprising cholesterol-5,6-epoxide hydrolase enzymes involved in cholesterol biosynthesis. SERMs including tamoxifen inhibit these enzymes leading to accumulation of sterol precursors—triggering autophagic survival [59]. By delaying apoptosis or DNA damage mechanisms, autophagy can be viewed as an adaptive response to promote the survival of the targeted tumour cells [29, 49, 60]. It has also been postulated that autophagy enables cancer cells to adopt a dormant state [18, 61]. Dormancy can ultimately lead to relapse and re-growth of cancer cells to facilitate their long-term survival. The underlying mechanisms of therapy resistance and tumour cell dormancy are not yet comprehensively understood but are likely to involve a state of cellular senescence, mediated by autophagy [26]. Molecular understanding of the functional roles of autophagy in tumour maintenance and resistance to environmental and external stresses will guide targeting autophagy. Indeed, evidence accumulates appearing to justify the development of autophagy inhibitors as a strategy in cancer therapy.

## Clinical developments: autophagy inhibition therapies

All autophagy modulating drugs in clinical trials are inhibitors of the process, primarily seeking to increase tumour sensitivity to conventional therapies [29]. It is widely accepted that autophagy inhibition could help sensitise cancer cells to cytotoxic therapies and potentiate the effects of current treatments in the clinic.

Chloroquine and its derivatives, previously used as anti-malarial agents, are leading the field and are currently the only FDA-approved modulators of autophagy [62]. Pre-clinical evidence has demonstrated the effectiveness of inhibiting autophagy to enhance chemotherapy cytotoxicity; this has been achieved either as a monotherapy or in combination with current chemotherapeutic drugs [62]. Chloroquine and its derivatives limit the acidification of lysosomes to inhibit their role in autophagic degradation [62–64]. Hydroxychloroquine is the preferred analogue because of its enhanced potency and limited side effects; the current phase I and phase II clinical trials in place involving hydroxychloroquine in combination with a variety of anti-cancer agents possessing different modes of action and used to treat distinct cancer phenotypes are detailed in Table 1 [46, 62, 65].

**Table 1:** Current autophagy inhibitors in clinical trials

Drug 1	Drug 1	Mode of action		Phase
HCQ	As a single agent		ER + breast cancer	I
HCQ	As a single agent		Prostate cancer	II
HCQ	Sunitinib	Tyrosine kinase inhibitor	Adult solid neoplasm	I
HCQ	Vorinostat	Histone deacetylase inhibitor	Malignant solid tumour	I
HCQ	Vorinostat	Histone deacetylase inhibitor	Colorectal cancer	I/II
HCQ	Vorinostat/sirolimus	Histone deacetylase inhibitor/mTOR inhibitor	Advanced cancers	I
HCQ	MKK0226	Akt inhibitor	Advanced cancers	I
HCQ	Gemcitabine	Anti-metabolite	Advanced adenocarcinoma	I/II
HCQ	Gemcitabine/carboplatin	Anti-metabolite/alkylating agent	Small cell lung cancer	I/II
HCQ	Interleukin-2	Immune modulator	Renal cell carcinoma	I/II
HCQ	Capecitabine	Anti-metabolite	Pancreatic carcinoma	II
HCQ	Abraxane and gemcitabine	Anti-microtubule agent/anti-metabolite	Pancreatic carcinoma	II

There are several phase I and phase II clinical trials for hydroxychloroquine in combination with many anti-cancer treatments in different cancer phenotypes [46, 62, 65]

**Fig. 5:**
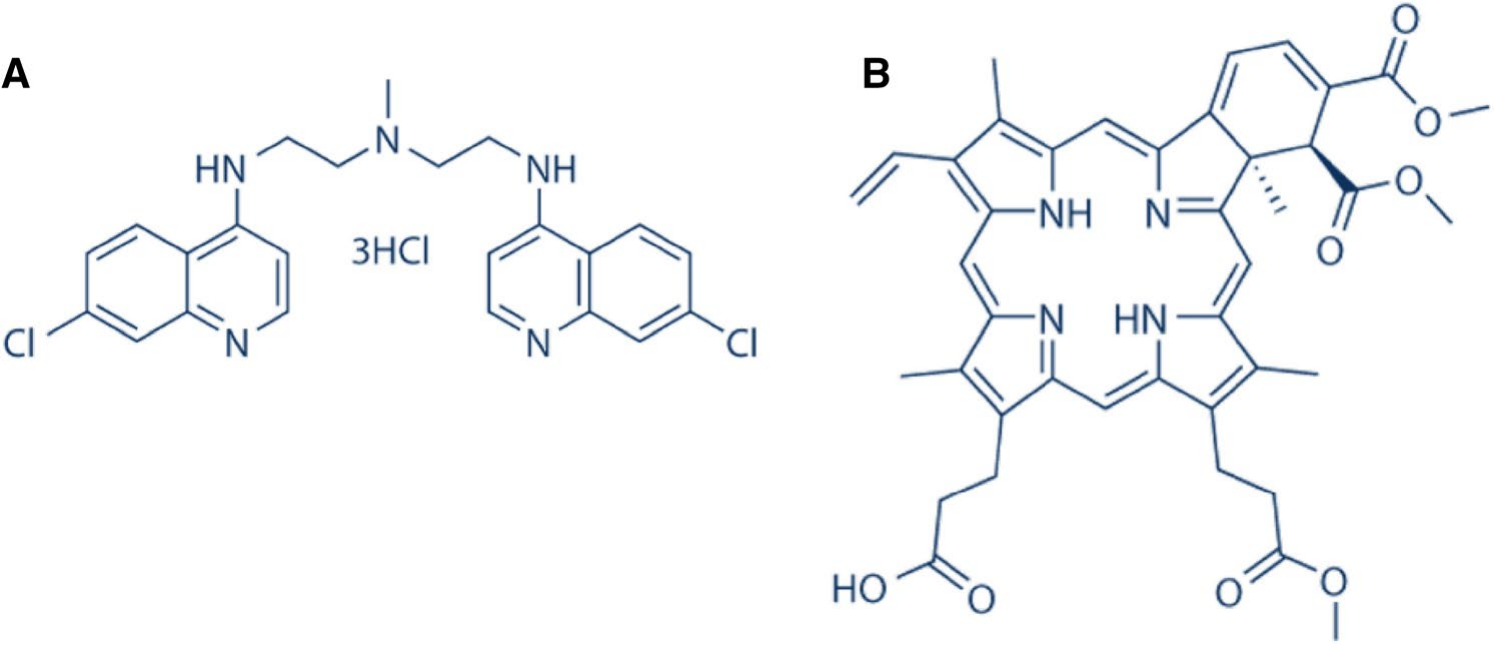
Structures of autophagy inhibitors: **a** Lys05; **b** verteporfin

Although clinical trials for hydroxychloroquine appear promising in both solid and haematological malignancies, the requirement for more specific and potent inhibitors remains. Lys05, a bisaminoquinoline derivative of chloroquine (Fig. 5a), has led to more pronounced lysosomal accumulation and deacidification resulting in impaired autophagy and tumour growth inhibition; moreover, single agent antitumor activity was observed without toxicity [66]. Mice were observed to develop an intestinal phenotype resembling that of mice and humans with genetic defects in the autophagy gene *Atg16L1*. Together these data provide evidence that Lys05 targets autophagy, and validate the therapeutic potential of this agent [64, 67]. Verteporfin (Fig. 5b) is another novel drug which acts at an earlier stage of the process to prevent initial formation of the autophagosome. In combination with the anti-metabolite gemcitabine, verteporfin has been shown to reduce pancreatic tumour growth in a pre-clinical setting [68]. This highlights the fact that autophagy inhibition can be targeted at multiple stages of the process. It can thus be argued that autophagy modulation presents an attractive therapeutic concept, particularly in regards to potentiation of anti-tumour activity and sensitisation of therapy-resistant tumours.

## Summary

To summarise, increasing our understanding of the processes of autophagy and its roles in different stages of cancer will underpin developments in this field. Evidence primarily supports autophagy as a tumour suppressive mechanism, with autophagy-related genes commonly deleted or defective in different cancer types. Autophagy suppresses tumour initiation by preventing inflammation and oncogenic proliferation. Maintenance of genomic stability is also a key property of autophagy tumour suppression and is most likely achieved by the prevention of damaged mitochondrial accumulation and subsequent generation of oxidative stress. Autophagy and immune-surveillance are intimately linked to prevent tumourigenesis. As an alternative form of programmed cell death, autophagy has been implicated as a mechanism of cytotoxicity in current anti-cancer treatments.

Thus, it is of therapeutic interest to induce autophagy to enhance tumour prevention or elimination. Such a strategy could be beneficial in patients with increased risk of developing cancer, particularly in cancer types with known and defined pre-cancerous evolution. Identifying biomarkers, such as p62, could help to identify autophagy-deficient patients who would best respond to this form of treatment.

Inducing autophagy in apoptosis-resistant cells could provide an alternative route of controlled cancer cell death, which could be crucial in surmounting therapy resistance. Furthering our understanding of the interactions between apoptosis, autophagy and other forms of cell death will be the key in developing alternative mechanisms towards targeting cancer cell toxicity.

The elusive autophagic switch from tumour suppressor to tumour promoter is evident once tumours are established. Cancer cells exploit autophagy to sustain increased metabolic demands and survive under various stresses including hypoxia, acidity and anti-cancer therapies. This poses one of the key challenges in the clinic: therapy resistance. With this in mind, autophagy inhibition poses an attractive therapeutic strategy, with clinical advances already taking place using a combinatorial approach with hydroxychloroquine in phase I and II trials. The important role of autophagy in the anti-cancer immune response must also be considered; autophagy inhibition could be combined with immune-stimulatory drugs, such as checkpoint inhibitors, to prevent immune down-regulation [69]. It is anticipated in the future that autophagy inhibition may help to sensitise resistant cancer cells to conventional therapies already in place. Specific cancer types which are ‘autophagy-addicted’ are most likely to benefit from autophagy inhibitors, indicating the potential wide application of these drugs.

To conclude, the role of autophagy in cancer is complex, context-dependent and far from fully elucidated.

Under normal physiology, autophagy can be a suppressor of tumourigenesis—a mean of ridding the body of damaged cells and ‘recycling’ nutrients. There is a role for autophagy in immune-surveillance and inhibition of malignant transformation; however, paradoxically, autophagy may support progression of established tumours, as the process can be subverted to ‘feed’ tumour cells. Autophagy may contribute to chemotherapy resistance, therefore its inhibition may sensitise cancer cells to therapy; yet the importance of chemotherapy-induced cytotoxic autophagy is evident, specifically in apoptosis-resistant tumour cells. Evidence supporting each of these statements has been discussed in this review and must be considered if, or when autophagy is to be exploited in a therapeutic setting. Fully understanding the autophagic switch will help to expose novel targets and mechanisms to target cancer cells.

In the future, it is likely that autophagy modulation will be specific and tailored to different cancer phenotypes, depending on tumour evolution and autophagy-dependency.

Today, autophagy exemplifies a controversial issue and an intense area of research; nevertheless, its modulation potentially represents an exciting novel approach in cancer treatment.

## Abbreviations

*Atg* genes: Autophagy-related genes
mTOR: Mammalian target of rapamycin
PI3K: Phosphatidylinositol-3-kinase
ROS: Reactive oxygen species
DDA: Dendrogenin A
LXR: Liver X receptor
LC3: Microtubule-associated protein 1A/1B-light chain 3
TFEB: Transcription factor EB
TAMR: Tamoxifen-resistant
HDAC: Histone deacetylase
SAHA: Suberoylanilide hydroxamic acid
GBM: Glioblastoma multiforme
V600E: Valine to glutamic acid mutation at amino acid 600 of BRAF
SERM: Selective oestrogen receptor modulator
ER: Oestrogen receptor
FDA: Food and drug administration

## References

[1] Mizushima N, Levine B, Cuervo AM, Klionsky DJ (2008). NIH public access. Nature.

[2] Sabatini DD, Adesnik M (2013). Christian de Duve: explorer of the cell who discovered new organelles by using a centrifuge. Proc Natl Acad Sci.

[3] Yang Z, Klionsky D (2010). Regulation. Curr Opin Cell Biol.

[4] White E (2015). The role for autophagy in cancer (White, 2015).pdf. J Clin Invest.

[5] Rebecca VW, Amaravadi RK (2016). Emerging strategies to effectively target autophagy in cancer. Oncogene.

[6] Cheng Y, Ren X, Hait WN, Yang J-M (2013). Therapeutic targeting of autophagy in disease: biology and pharmacology. Pharmacol Rev.

[7] Lum JJ, DeBerardinis RJ, Thompson CB (2005). Autophagy in metazoans: cell survival in the land of plenty. Nat Rev Mol Cell Biol.

[8] Talloczy Z, Jiang W, Virgin HW, th (2002). Regulation of starvation- and virus-induced autophagy by the eIF2alpha kinase signaling pathway. Proc Natl Acad Sci USA.

[9] Manent J, Banerjee S, De Matos Simoes R (2017). Autophagy suppresses Ras-driven epithelial tumourigenesis by limiting the accumulation of reactive oxygen species. Oncogene.

[10] Botti J, Djavaheri-Mergny M, Pilatte Y, Codogno P (2006). Autophagy signaling and the cogwheels of cancer. Autophagy.

[11] Liang XH, Jackson S, Seaman M (1999). Induction of autophagy and inhibition of tumorigenesis by beclin 1. Nature.

[12] Aita VM, Liang XH, Murty VV (1999). Cloning and genomic organization of beclin 1, a candidate tumor suppressor gene on chromosome 17q21. Genomics.

[13] Koneri K, Goi T, Hirono Y (2007). Beclin 1 gene inhibits tumor growth in colon cancer cell lines. Anticancer Res.

[14] Miracco C, Cosci E, Oliveri G (2007). Protein and mRNA expression of autophagy gene Beclin 1 in human brain tumours. Int J Oncol.

[15] Kang R, Zeh HJ, Lotze MT, Tang D (2011). The Beclin 1 network regulates autophagy and apoptosis. Cell Death Differ.

[16] Marquez RT, Xu L (2012). Bcl-2:Beclin 1 complex: multiple, mechanisms regulating autophagy/apoptosis toggle switch. Am J Cancer Res.

[17] Takamura A, Komatsu M, Hara T (2011). Autophagy-deficient mice develop multiple liver tumors. Genes.

[18] Mathew R, Karantza-Wadsworth V, White E (2007). Role of autophagy in cancer. Nat Rev Cancer.

[19] Kihara A, Kabeya Y, Ohsumi Y, Yoshimori T (2001). Beclin-phosphatidylinositol 3-kinase complex functions at the trans-Golgi network. EMBO Rep.

[20] Kihara A, Noda T, Ishihara N, Ohsumi Y (2001). Two distinct Vps34 phosphatidylinositol 3-kinase complexes function in autophagy and carboxypeptidase y sorting in *Saccharomyces cerevisiae*. J Cell Biol.

[21] Hanahan D, Weinberg RA (2011). Hallmarks of cancer: the next generation. Cell.

[22] Lamark T, Kirkin V, Dikic I, Johansen T (2009). NBR1 and p62 as cargo receptors for selective autophagy of ubiquitinated targets. Cell Cycle.

[23] White E, Mehnert JM, Chan C (2015). Autophagy, metabolism, and cancer. Clin Cancer Res.

[24] Mathew R, Karp C, Beaudoin B, Vuong N, Chen G, Chen H-Y, Bray K, Reddy A, Bhanot G, Gelinas C, DiPaola RS, Karantza-Wadsworth V, White E (2009). Autophagy suppresses tumorigenesis through elimination of p62. Cell.

[25] Yang A, Rajeshkumar NV, Wang X, Yabuuchi S, Alexander BM, Chu GC, Von Hoff DD, Maitra A, Kimmelman A (2014). Autophagy is critical for pancreatic tumor growth and progression in tumors with p53 alterations. Cancer Discov.

[26] Nagelkerke A, Sweep FCGJ, Geurts-Moespot A (2015). Therapeutic targeting of autophagy in cancer. Part I: Molecular pathways controlling autophagy. Semin Cancer Biol.

[27] Michaud M, Martins I, Sukkurwala AQ, Adjemian S, MA YT, Pellagati P, Shen SS, Keppo O, Scoazec M, Mignot G, Rello-Varona S, Tailler M, Menger L, Vacchelli E, Galluzzi L, Ghiringheli F, di Virgilio F, Zitvogel L, Kroemer G (2011). Autophagy-dependent anticancer immune responses induced by chemotherapeutic agents in mice. Science.

[28] Pietrocola F, Bravo-San Pedro JM, Galluzzi L, Kroemer G (2017). Autophagy in natural and therapy-driven anticancer immunosurveillance. Autophagy.

[29] Pietrocola F, Pol J, Vacchelli E (2016). Autophagy induction for the treatment of cancer. Autophagy.

[30] Ko A, Kanehisa A, Martins I (2014). Autophagy inhibition radiosensitizes in vitro, yet reduces radioresponses in vivo due to deficient immunogenic signalling. Cell Death Differ.

[31] Galluzzi L, Pedro JMBS, Demaria S (2017). Activating autophagy to potentiate immunogenic chemotherapy and radiation therapy. Nat Rev Clin Oncol.

[32] Levine B, Yuan J (2005). Autophagy in cell death: an innocent convict?. J Clin Invest.

[33] Galluzzi L, Vitale I, Abrams JM (2012). Molecular definitions of cell death subroutines: recommendations of the Nomenclature Committee on Cell Death 2012. Cell Death Differ.

[34] Segala G, David M, De Medina P (2017). Dendrogenin A drives LXR to trigger lethal autophagy in cancers. Nat Commun.

[35] de Medina P, Paillasse MR, Segala G, Voisin M, Mhamdi L, Dalenc F, Lacroix-Triki M, Filleron T, Pont F, Al Saati T, Morisseau C, Hammock BD, Silvente-Poirot S, Poirot M (2013). Dendrgenin A arises from cholesterol and histamine metabolism and shows cell differentiation and anti-tumour properties. Nat Commun.

[36] Poirot M, Silvente-Poirot S (2018). The tumor-suppressor metabolite, dendrogenin A, is a new class of LXR modulator activating lethal autophagy in cancers. Biochem Pharmacol.

[37] Silvente-Poirot S, Segala G, Poirot MC, Poirot M (2018). Ligand-dependent transcriptional induction of lethal autophagy. Autophagy.

[38] Opipari AW, Tan L, Boitano AE (2004). Resveratrol-induced autophagocytosis in ovarian cancer cells resveratrol-induced autophagocytosis in ovarian cancer cells. Cancer Res.

[39] Kanzawa T, Kondo Y, Ito H (2003). Induction of autophagic cell death in malignant glioma cells by arsenic trioxide. Cancer Res.

[40] Bursch W, Ellinger A, Kienzl H, Torok L, Pandey S, Sikorska M, Walker R, Hermann R (1996). Active cell death induced by the anti-estrogens tamoxifen and ICI 164 384 in human mammary carcinoma cells (MCF-7) in culture: the role of autophagy. Carcinogenesis.

[41] Lee YJ, Won AJ, Lee J (2012). Molecular mechanism of SAHA on regulation of autophagic cell death in tamoxifen-resistant MCF-7 breast cancer cells. Int J Med Sci.

[42] Zhang J, Hummersone M, Matthews CS (2015). N3-substituted temozolomide analogs overcome methylguanine-DNA methyltransferase and mismatch repair precipitating apoptotic and autophagic cancer cell death. Oncology.

[43] Bussell K (2004). Cell death—death by self-digestion. Nat Rev Mol Biol.

[44] Strohecker AM, White E (2014). Targeting mitochondrial metabolism by inhibiting autophagy in Braf-driven cancers. Cancer Discov.

[45] Amaravadi R, Lippincott-Schwartz J, Yin X-M (2011). Principles and current strategies for targeting autophagy for cancer treatment. Clin Cancer.

[46] Galluzzi L, Bravo-San Pedro JM, Vitale I (2015). Essential versus accessory aspects of cell death: recommendations of the NCCD 2015. Cell Death Differ.

[47] Sehgal AR, Konig H, Johnson DE, Tang D, Amaravadi RK, Boyiadzis M, Lotze M (2015). You eat what you are: autophagy inhibition as a therapeutic strategy in leukemia. Leukemia.

[48] Rouschop KMA, Wouters B (2009). Regulation of autophagy through multiple independent hypoxic signaling pahways. Curr Mol Med.

[49] Song JR, Qu ZQ, Guo XL, Zhao QD, Gao L, Sun K, Shen F, Wu MC, Wei L (2009). Hypoxia-induced autophagy contributes to the chemoresistance of hepatocellular carcinoma cells. Autophagy.

[50] Guo JY, Karsli-Uzunbas G, Mathew R (2013). Autophagy suppresses progression of K-ras-induced lung tumors to oncocytomas and maintains lipid homeostasis. Genes Dev.

[51] Kim MJ, Woo SJ, Yoon CH (2011). Involvement of autophagy in oncogenic K-Ras-induced malignant cell transformation. J Biol Chem.

[52] Guo JY, Chen HY, Mathew R (2011). Activated Ras requires autophagy to maintain oxidative metabolism and tumorigenesis. Genes Dev.

[53] Mancias JD, Kimmelman AC (2011). Targeting autophagy addiction in cancer. Oncotarget.

[54] Rosenfeldt MT, O’Prey J, Morton JP (2013). P53 status determines the role of autophagy in pancreatic tumour development. Nature.

[55] Lock R, Roy S, Kenific CM (2011). Autophagy facilitates glycolysis during Ras-mediated oncogenic transformation. Mol Biol Cell.

[56] Yang S, Wang X, Contino G (2011). Pancreatic cancers require autophagy for tumor growth. Genes Dev.

[57] Strohecker AM, White E (2014). Targeting mitochondrial metabolism by inhibiting autophagy in BRAF-driven cancers. Cancer Discov.

[58] De Medina P, Silvente-Poirot S, Poirot M (2009). Tamoxifen and AEBS ligands induced apoptosis and autophagy in breast cancer cells through the stimulation of sterol accumulation. Autophagy.

[59] Leignadier J, Dalenc F, Poirot M, Silvente-Poirot S (2017). Improving the efficacy of hormone therapy in breast cancer: the role of cholesterol metabolism in SERM-mediated autophagy, cell differentiation and death. Biochem Pharmacol.

[60] Abedin MJ, Wang D, McDonnell MA (2007). Autophagy delays apoptotic death in breast cancer cells following DNA damage. Cell Death Differ.

[61] Lu Z, Luo RZ, Lu Y (2008). The tumor suppressor gene ARHI regulates autophagy and tumour dormancy in human ovarian cancer cells. Cell Prolif.

[62] Chude CI, Amaravadi RK (2017). Targeting autophagy in cancer: update on clinical trials and novel inhibitors. Int J Mol Sci.

[63] Wang Y, Peng RQ, Li DD (2011). Chloroquine enhances the cytotoxicity of topotecan by inhibiting autophagy in lung cancer cells. Chin J Cancer.

[64] Pellegrini P, Strambi A, Zipoli C (2014). Acidic extracellular pH neutralizes the autophagy-inhibiting activity of chloroquine: implications for cancer therapies. Autophagy.

[65] Amaravadi RK, Yu D, Lum JJ (2007). Autophagy inhibition enhances therapy-induced apoptosis in a Myc-induced model of lymphoma. J Clin Invest.

[66] Amaravadi RK, Winkler JD (2012). Lys05: a new lysosomal autophagy inhibitor. Autophagy.

[67] McAfee Q, Zhang Z, Samanta A (2012). Autophagy inhibitor Lys05 has single-agent antitumor activity and reproduces the phenotype of a genetic autophagy deficiency. Proc Natl Acad Sci.

[68] Donohue E, Thomas A, Maurer N (2013). The autophagy inhibitor verteporfin moderately enhances the antitumor activity of gemcitabine in a pancreatic ductal adenocarcinoma model. J Cancer.

[69] Duffy A, Le J, Sausville E, Emadi A (2015). Autophagy modulation: a target for cancer treatment development. Cancer Chemother Pharmacol.

